# Correlation of High Magnetoelectric Coupling with Oxygen Vacancy Superstructure in Epitaxial Multiferroic BaTiO_3_-BiFeO_3_ Composite Thin Films

**DOI:** 10.3390/ma9010044

**Published:** 2016-01-13

**Authors:** Michael Lorenz, Gerald Wagner, Vera Lazenka, Peter Schwinkendorf, Michael Bonholzer, Margriet J. Van Bael, André Vantomme, Kristiaan Temst, Oliver Oeckler, Marius Grundmann

**Affiliations:** 1 Institut für Experimentelle Physik II, Universität Leipzig, Leipzig D-04103, Germany; schwinkendorf@physik.uni-leipzig.de (P.S.); bonholzer@physik.uni-leipzig.de (M.B.); grundmann@physik.uni-leipzig.de (M.G.); 2 Institut für Mineralogie, Kristallographie und Materialwissenschaft, Universität Leipzig, Leipzig D-04103, Germany; wagner@chemie.uni-leipzig.de (G.W.); oliver.oeckler@uni-leipzig.de (O.O.); 3 Instituut voor Kern- en Stralingsfysica, KU Leuven, Leuven B-3001, Belgium; vera.lazenka@fys.kuleuven.be (V.L.); andre.vantomme@fys.kuleuven.be (A.V.); kristiaan.temst@fys.kuleuven.be (K.T.); 4 Laboratorium voor Vaste-Stoffysica en Magnetisme, KU Leuven, Leuven B-3001, Belgium; margriet.vanbael@fys.kuleuven.be

**Keywords:** oxide thin films, multiferroic composites, magnetoelectric coupling, magnetoelectric voltage coefficient, oxygen vacancy superstructure, pulsed laser deposition

## Abstract

Epitaxial multiferroic BaTiO_3_-BiFeO_3_ composite thin films exhibit a correlation between the magnetoelectric (ME) voltage coefficient α_ME_ and the oxygen partial pressure during growth. The ME coefficient α_ME_ reaches high values up to 43 V/(cm·Oe) at 300 K and at 0.25 mbar oxygen growth pressure. The temperature dependence of α_ME_ of the composite films is opposite that of recently-reported BaTiO_3_-BiFeO_3_ superlattices, indicating that strain-mediated ME coupling alone cannot explain its origin. Probably, charge-mediated ME coupling may play a role in the composite films. Furthermore, the chemically-homogeneous composite films show an oxygen vacancy superstructure, which arises from vacancy ordering on the {111} planes of the pseudocubic BaTiO_3_-type structure. This work contributes to the understanding of magnetoelectric coupling as a complex and sensitive interplay of chemical, structural and geometrical issues of the BaTiO_3_-BiFeO_3_ composite system and, thus, paves the way to practical exploitation of magnetoelectric composites.

## 1. Introduction

Multiferroic composites consisting of two different chemical compounds offer unique flexibility in geometrical and structural design to achieve desired functional properties, in particular a high magnetoelectric coupling [1,2]. Correlations at the interfaces and novel approaches in combining magnetism and ferroelectricity are mentioned by Fiebig and Spaldin as outlooks in the research on novel magnetoelectrics [3]. Progress reports highlight the impressive variability of multiferroic composite materials, which in the future should also allow implementation of electrical transport into the multiferroic concept [4,5]. Kleemann *et al.* discussed the complex magnetoelectric (ME) effects of single-phase Type I (such as BiFeO_3_) and Type II (such as TbMnO_3_) multiferroics in comparison to multiphase composites and higher-order ME effects in disordered Type III multiferroics [6]. For a discussion of possible units of the ME coefficient α_ME*ij*_ = *E_i_*/*H_j_*, see [7]. Giant magnetoelectric coefficients α_ME_ are reported in two-two composite multilayers consisting of AlN and amorphous (Fe_90_Co_10_)_78_Si_12_B_10_ on Si(100). The α_ME_ values reach 737 V/(cm·Oe) at a mechanical resonance of 753 Hz and 3.1 V/(cm·Oe) out of a resonance at 100 Hz [8]. These AlN-(Fe_90_Co_10_)_78_Si_12_B_10_ composites are applied as magnetic field sensors. The consideration of exchange bias coupling allows a tuning of the detection limit and ME coefficient of these sensors [9].

Little work is reported on composites containing BiFeO_3_; some papers mostly on bulk solid solutions are discussed in the review [10] and in [11]. The coexistence of ferroelectricity and ferromagnetism in BiFeO_3_-BaTiO_3_ thin films at room temperature was reported already in 1999 [12]. More recently, the group around Ramesh combined the multiferroic BiFeO_3_ with ferrimagnetic CoFe_2_O_4_ in self-organized nanostructures with a 300-nm total thickness and obtained a transverse ME susceptibility of 60 mV/(cm·Oe) [13]. Muragavel *et al*. have grown multiferroic epitaxial (BiFeO_3_)*_x_*-(BaTiO_3_)_(1−*x*)_ composite films (*x* = 0.3–0.9) on SrTiO_3_:Nb and found compressive strain for *x* < 0.7 and relaxed growth for *x* > 0.7 and a corresponding phase change from rhombohedral to tetragonal [14]. Epitaxial Co- and Fe-substituted BiFeO_3_ films for spin-filter applications are reported in [15]. Mößbauer spectroscopy was applied to bulk (BiFeO_3_)*_x_*-(BaTiO_3_)_(1−*x*)_ ceramics (*x* = 0.9–0.7), and relatively low α_ME_ values up to 0.6 mV/(cm·Oe) were obtained (1 kHz, H_DC_ = 0) [16]. Nearly a complete mixing ratio of polycrystalline bulk (BiFeO_3_)*_x_*-(BaTiO_3_)_(1−*x*)_ ceramics (*x* = 0.025–1) was investigated, and a maximum α_ME_ of 0.87 mV/(cm·Oe) was found for *x* = 0.725 [17]. Compositionally-modulated (Co/Mg/Ni)Fe_2_O_4_ spinel nanopillars embedded in a BiFeO_3_ matrix film have been reported recently together with their magnetic response [18]. Reviews on the properties and device applications of the single-phase multiferroic compound BiFeO_3_ are published in [19,20]. We reported the effect of rare-earth doping on the multiferroic properties of BiFeO_3_ thin films [21].

In continuation of this latter work on BiFeO_3_, we found that the ME coefficient of single-phase BiFeO_3_ thin films can be considerably enhanced by combination with the ferroelectric BaTiO_3_ in both superlattices and in chemically-homogeneous composite thin films [11,22,23]. In particular, while a BiFeO_3_ film showed an α_ME_ of about 2 V/(cm·Oe), the corresponding (BaTiO_3_-BiFeO_3_) × 15 superlattice showed 9 V/(cm·Oe) at 300 K, clearly demonstrating the interface effect on the magnetic moment direction and magnetoelectric coupling, as published in [23]. In our first chemically-homogeneous composite films published in [11], we combined the ferroelectric BaTiO_3_ and multiferroic BiFeO_3_ phase into a thin film nanocomposite structure, and the detailed connectivity scheme and coupling mechanism in terms of the microscopic origin of the measured high ME coefficients α_ME_ up to 21 V/(cm·Oe) have not been clear up to now.

Therefore, we present in this paper a more detailed investigation of the microstructure of the chemically-homogeneous BaTiO_3_-BiFeO_3_ composite films based on scanning transmission electron microscopy (STEM) and selected area electron diffraction (SAED). Furthermore, we discuss the optimum oxygen supply during the growth of the epitaxial composite films to achieve the maximum magnetoelectric voltage coefficient and the corresponding film structure, as well as probable magnetoelectric coupling mechanisms. In the following, we provide results on the solid solution BaTiO_3_-BiFeO_3_ composite films. The BaTiO_3_-BiFeO_3_ superlattice heterostructures with clear spatial separation of both phases as published in [11,22,23] are mentioned in the discussion only for a comparison.

## 2. Results and Discussion

### 2.1. Out-of-Plane Strain and Crystalline Structure

In agreement with the modeling of the magnetoelectric voltage coefficient in dependence of the volume fraction of the piezoelectric component in various lead-based and lead-free oxide composites [1,24], we have chosen the source target composition for our composite film growth by pulsed laser deposition (PLD) to be 67 wt % BaTiO_3_ and 33 wt % BiFeO_3_. The oxygen partial pressure during PLD was varied between 0.01 mbar and 0.5 mbar. As the growth rate of the composite films depends strongly on the oxygen partial pressure, it is natural that the film thickness of the samples is not uniform. We used several sample series for the results presented here (see the running sample numbers in Table 1). In the last series (G5556–G5562), we tried to compensate for the changing growth rate by using different total numbers of laser pulses for the growth, resulting in reduced thickness variation from 208–388 nm only, instead of up to 1000 nm in the previous series. The substrate material of the films intended for X-ray diffraction (XRD) and ferroelectric and magnetoelectric measurements was SrTiO_3_:Nb(001). For the STEM and SAED investigations only, films grown simultaneously on MgO(001) were used; see the Experimental Section for further insight into our growth regime. For oxygen-deficient weakly-Mn-doped ZnO thin films, we have recently measured the trigonal distortion of oxygen tetrahedra; see [25]. Thus, lower oxygen partial pressure in PLD growth is clearly correlated to increasing density of oxygen vacancies. With that, we are able to correlate the ME coefficient to the oxygen deficiency of the composites and related structural properties.

**Table 1:** X-ray diffraction (XRD) lattice parameters of SrTiO_3_ substrates and BaTiO_3_-BiFeO_3_ composite films on SrTiO_3_:Nb(001) at the indicated growth pressures p(O_2_). Composite film thickness was determined at focused ion beam (FIB) cross-sections by scanning transmission electron microscopy (STEM) for one sample of each growth series. *c*-lattice parameters were calculated from wide-angle 004 peaks. The bulk lattice parameters are as follows (nm): SrTiO_3_: *a* = 0.3905 (JCPDS 84-0444); BaTiO_3_: *a* = 0.39945, *c* = 0.40335 (JCPDS 83–1880); BiFeO_3_: *a* = 0.3962 (JCPDS 73–0548). The out-of-plane strain values Δ*c*/*c*_0_ are calculated assuming an averaged bulk lattice parameter of the 67 wt % BaTiO_3_-33 wt % BiFeO_3_ composite of *c*_0_ = 0.40099 nm. For visualization of the *c*_film_ and FWHM(ω) values, see Figure 1b, Figure 2 and Figure 3, respectively. The accuracy of the lattice parameters is about ±0.0001 nm. Several samples for each growth pressure are included in the table to get an impression about the reproducibility of pulsed laser deposition (PLD) and the accuracy of lattice parameters. The sample numbers show which films were grown simultaneously (identical No.) or in consecutive PLD runs (successive No.), respectively.

p(O_2_) (mbar)	Sample No.	Thickness (nm)	*c*_substrate_ (nm)	*c*_film_ (nm)	Strain (*c* − *c*_0_)/*c*_0_	FWHM(ω) $\bar{1}03$ (°)
0.01	G 5084b	730	0.39055	0.41030	2.322%	0.127
0.01	G 5084c	730	0.39066	0.40991	2.224%	0.125
0.1	G 5085c	488	0.39066	0.40708	1.519%	0.109
0.1	G 5562b	208	0.39067	0.40767	1.666%	0.734 *
0.1	G 5562d	208	0.39081	0.40618	1.294%	0.157
0.15	G 5561b	347	0.39067	0.40593	1.232%	0.162
0.15	G 5561d	347	0.39069	0.40554	1.135%	0.320 *
0.2	G 5560b	365	0.39068	0.40403	0.758%	0.131
0.25	G 4728d	285	0.39069	0.40103	0.010%	0.237
0.25	G 5559b	388	0.39067	0.40168	0.172%	0.272
0.325	G 5558b	352	0.39068	0.40148	0.122%	0.303
0.4	G 5557d	311	0.39070	0.40148	0.122%	0.354
0.5	G 5086b	1000	0.39056	0.40133	0.085%	0.346
0.5	G 5556b	256	0.39068	0.40150	0.127%	0.354

* Extra peak broadening due to substrate domains; see Figure S1.

**Figure 1:**
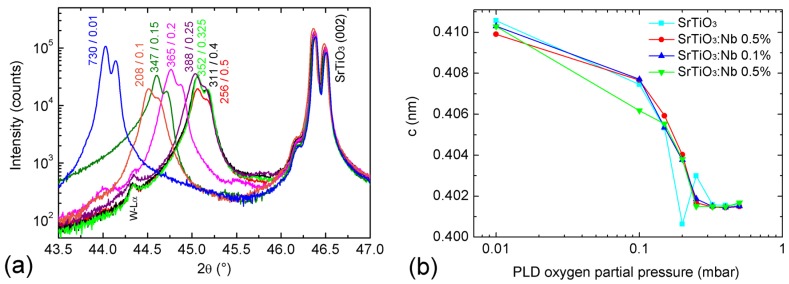
(**a**) X-ray diffraction (XRD) 2θ-ω scans of BaTiO_3_-BiFeO_3_ composite thin films grown on SrTiO_3_:Nb(001) at the indicated oxygen partial pressures. The two numbers at the film peaks indicate the total composite film thickness in nm, and the oxygen partial pressure during pulsed laser deposition (PLD) growth, respectively. Note the Kα_1/2_ splitting of each peak. W-Lα is a spectral line from the X-ray tube. The single-phase contributions of BaTO_3_ and BiFeO_3_ cannot be resolved here; see Table 1. (**b**) PLD oxygen pressure evolution of the *c*-axis lattice parameters calculated from 004 peaks of four BaTiO_3_-BiFeO_3_ composite thin films grown simultaneously for each growth pressure at the indicated substrates. See Table 1 for the values of the c-lattice parameters and more structural details.

**Figure 2:**
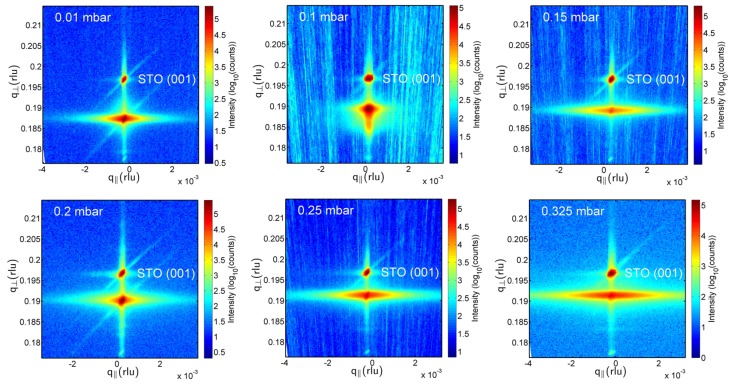
XRD reciprocal space maps around the symmetric SrTiO_3_ 001 peaks of BaTiO_3_ (67 wt %)-BiFeO_3_ (33 wt %) composite thin films grown at the indicated PLD oxygen partial pressures. The separation of a point-like substrate and a broadened composite film peak decreases with increasing oxygen partial pressure due to decreasing out-of-plane strain; see Table 1. The horizontal broadening of film peaks is a measure of the tilt mosaicity of the composites. STO stands for SrTiO_3_.

Figure 1a shows XRD 2θ-ω scans of the investigated BaTiO_3_-BiFeO_3_ composite films grown on SrTiO_3_:Nb(001) single crystalline substrates around the 002 peaks. Figure 1a, as well as the 001 reciprocal space maps (RSMs) in Figure 2 demonstrate the film peak shift to lower 2θ angles, *i.e.*, increasing out-of-plane lattice parameter with decreasing oxygen partial pressure. In Figure 1b, the corresponding *c*-lattice parameters as calculated from the positions of the 004 reflections of the films are plotted as depicted in Figure S2 in the Supplementary Materials. We found that using 004 peaks for the calculation of *c* values results in a smoother dependence on oxygen partial pressure in comparison to cos^2^θ extrapolations with omission of 001 or 001 + 002 peaks. Due to the goniometer height error, the accuracy of lattice parameters calculated from low-θ peaks, such as 001 and 002, is not sufficient for a precise analysis. In addition to Figure 2, the RSMs around the 001 peak in the Supplementary Materials show the impact of the tilt mosaicity of the SrTiO_3_ substrate on the composite film structure (Figure S1); and the impact of a changed 33 wt %-67 wt % BaTiO_3_-BiFeO_3_ composite film composition instead of the generally used 67 wt %-33 wt % on the film mosaicity (Figure S3).

As the lattice parameters of BaTiO_3_ and BiFeO_3_ as bulk and thin films are very close together (see the Joint Committee on Powder Diffraction Standards (JCPDS) data in the caption of Table 1), the XRD peaks of the BaTiO_3_ and BiFeO_3_ phases in our composites cannot be resolved. Even closer agreement of BaTiO_3_ and BiFeO_3_ lattice parameters is supported by [26], reporting for BiFeO_3_ in tetragonal *P*4*mm* symmetry *a* = 0.3935 and *c* = 0.3998 nm. Therefore, we assume an averaged out-of-plane lattice constant *c*_0_ to get estimates about the average out-of-plane strain of the composite films; see Table 1. A higher oxygen deficiency of the composite films results in increased out-of-plane strain. For a comparison, in our (BaTiO_3_-BiFeO_3_) × 15 superlattices, both phases could be separately detected by Rutherford backscattering spectrometry, XRD and STEM with nearly no intermixing at the interfaces [11,22].

**Figure 3:**
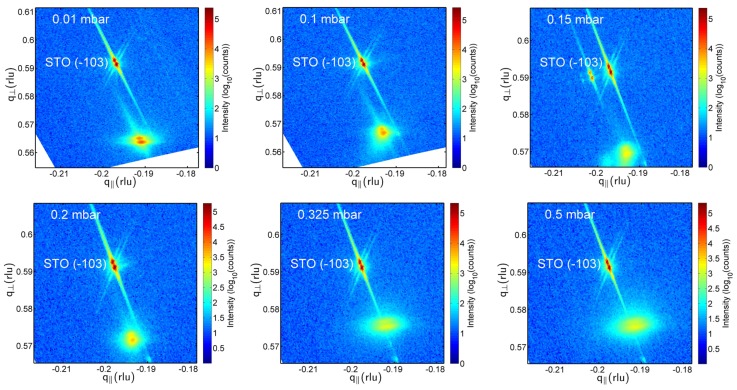
XRD reciprocal space maps around the asymmetric $\bar{1}03$ SrTiO_3_ substrate peaks (in the Figures referred to as STO (−103)) of the BaTiO_3_-BiFeO_3_ composite films grown at the indicated oxygen partial pressures. The lower intensity film peaks are at the bottom. With increasing oxygen pressure, the film peaks broaden, *i.e.*, the composite mosaicity increases. However, film-to-substrate peak separation decreases. Note that the vertical q_┴_ axis for the two lowest pressures is enlarged. As is visible from the increasing vertical misalignment of film and substrate peaks with increasing growth pressure, the film relaxation increases. The 0.15-mbar sample shows substrate and corresponding crystalline film domains. The peak splitting is due to the Kα_1/2_ radiation used.

Figure 3 shows typical RSMs around the asymmetric $\bar{1}03$ peaks of the composite films. As already visible in Figure 2, the film peaks broaden both horizontally and vertically with increasing oxygen pressure, *i.e.*, the film mosaicity and the variation of film lattice parameter increases. This broadening is quantitatively expressed in Table 1 by the FWHM(ω) values. From the increasing vertical misalignment of the film and substrate peaks with increasing growth pressure, we conclude an increasing film relaxation. That means that none of the grown composite films can be considered as in-plane lattice matched to the SrTiO_3_:Nb substrates. The in-plane a-lattice parameters as calculated using *c*_004_ (see Table 1) and the *d*-values extracted from the $\bar{1}03$ RSM peaks scatter around 0.404 ± 0.005 nm. Because of the limited accuracy of these in-plane lattice parameters, statements about in-plane strain cannot be derived from these values.

### 2.2. STEM and Oxygen Vacancy Superstructure

**Figure 4:**
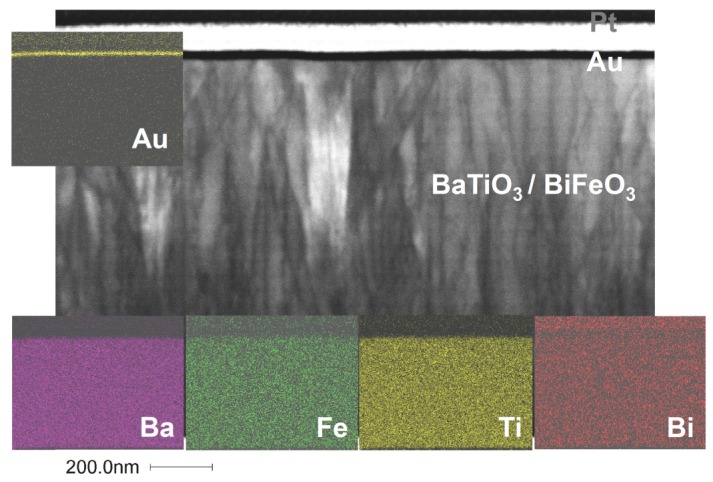
Scanning transmission electron microscopy (STEM) bright-field image of the BaTiO_3_-BiFeO_3_ composite film grown at 0.01 mbar oxygen pressure shown as the (110) cross-section. Clearly visible is the high density of dislocation lines in this particular composite film. The colored energy dispersive X-ray spectroscopy (EDX) maps demonstrate the homogeneous distribution of elements Ba, Fe, Ti and Bi in the plane of the cross-section. The gold film results from the extraction of the TEM cross-section out of the planar film sample using a focused ion beam. For more EDX maps and elemental analyses, see the Supplementary Materials, Figures S4, S6, S8 and S9.

**Figure 5:**
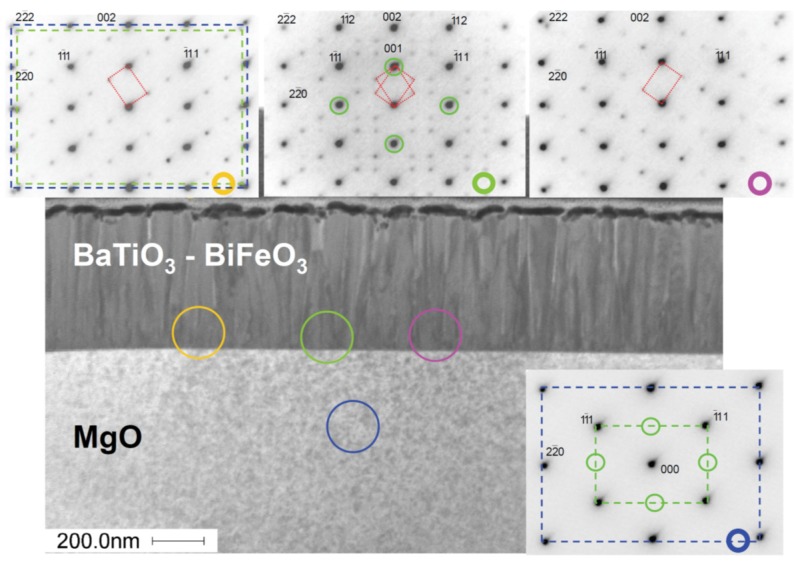
STEM dark-field image of the BaTiO_3_-BiFeO_3_ composite film grown at 0.25 mbar on MgO(001) taken from the (110) cross-section. The four selected area electron diffraction (SAED) patterns have been taken from the encircled regions at the interface or the substrate. The main spots from the composite (green circles) confirm the BaTiO_3_-type structure of the composite film. These are forbidden for MgO, as seen bottom right. The additional weak spots in the composite indicate superstructure reflections that are probably due to oxygen vacancy ordering. The red dotted lines show the two possible orientations of oxygen vacancy ordering.

Two composite film samples grown on MgO(001) were investigated by STEM and SAED, namely one sample grown at 0.01 mbar (see Figure 4 and Figures S4–S7) and the other at 0.25 mbar (see Figure 5 and Figures S7–S10). Because these films on MgO were grown simultaneously with the samples on SrTiO_3_ and SrTiO_3_:Nb, we expect the microstructural features found here to be representative of all of our BaTiO_3_-BiFeO_3_ composite thin films. Figure 4 shows a bright-field STEM micrograph of a BaTiO_3_-BiFeO_3_ composite thin film grown at the lowest pressure of 0.01 mbar. It shows a high density of dislocation lines in the film. Energy dispersive X-ray spectroscopy (EDX) maps taken at the STEM cross-sections indicate a homogeneous elemental distribution of Ba, Ti, Bi and Fe in the composite film. On top of the composite film is a thin gold and a platinum layer (Figure 4); see also Figures S4 and S8 for STEM dark field images and EDX maps of the 0.25-mbar and the 0.01-mbar samples, respectively.

In the dark-field STEM image of a cross-section of a BaTiO_3_-BiFeO_3_ composite film grown at 0.25 mbar in Figure 5, the columnar domains revealed by the grey scale contrast modulation reflect the different orientation of ordered oxygen vacancy layers, *i.e.*, ($\bar{1}11$) and/or ($1\bar{1}1$). Probably, the small microstrain differences between these domains are responsible for the high magnetoelectric coefficients α_ME_ reported below.

In Figure 5 (top), in the SAED patterns, the main reflections indicate the BaTiO_3_-type film structure of the 67 wt % BaTiO_3_-33 wt % BiFeO_3_ composite and the MgO structure of the substrate, which overlap. However, far away from the 000 reflection, a splitting appears due to small differences of the lattice parameters of MgO and BaTiO_3_-BiFeO_3_ composite film (see the blue and green frame in Figure 5, top left). Upon closer inspection of the film patterns in Figure 5, additional weaker reflections at ±⅓(*hkl*)_pseudocubic_ become visible, which indicate a superstructure with three-times the BaTiO_3_-BiFeO_3_ composite lattice parameter (Table 1). The superstructure clearly appears in two orientations, tilted left and right, and at some position, both orientations are observed simultaneously, such as in Figure 5, top center. The origin of the superstructure may be the same oxygen vacancy ordering, which is known from BaTiO_3_ grown in a reduced environment [27]. If the structural data (space group *P*3*m*1; see [27]) for such a “reduced BaTiO_3_” were used for SAED simulation, exactly the same pattern (also the weak reflections) appears at the same positions as seen experimentally. In “reduced BaTiO_3_”, every third of the hexagonal anion layers stacked along the pseudocubic <111> direction corresponds to a plane of ordered oxygen vacancies, *i.e.*, it remains “empty” [27].

**Figure 6:**
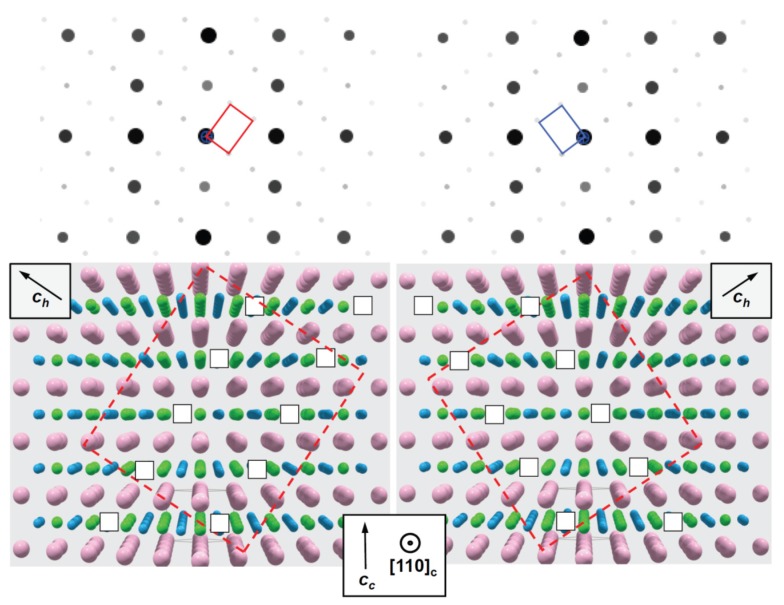
Structure model of oxygen vacancy ordering in planes parallel ($\bar{1}11$) (**left**) and ($1\bar{1}1$) (**right**), respectively; projection along [110] (directions according to cubic setting, subscript *c*; except the additionally indicated [001] direction with respect to the hexagonal setting, subscript *h*). The indication of atoms is as follows: blue, oxygen; pink, barium; green, titanium.

Several other types of perovskite superstructures have been discussed by Glazer [28] in order to explain additional weak reflections. Those in the SAED patterns shown in Figure 5 are consistent with the structure model shown in Figure 6. In addition, the oxygen vacancies involve the reduction of Ti^4+^ to Ti^3+^ as a consequence of charge neutrality, which is corroborated by electron energy loss spectroscopy [27]. Consistent with simple bond-valence considerations, the Ti^3+^ ions should be located in the incomplete oxygen atom octahedra. Thus, oxygen defects lead to ordered dipoles. Figures S5 and S7 in the Supplementary Materials show STEM and SAED images of the corresponding 0.01 mbar sample. There seems to be an indication of visually more intense oxygen superstructure reflections of the sample grown with higher oxygen deficiency, *i.e.*, at a lower pressure of 0.01 mbar. Figure S7 provides a direct comparison of these SAED images of both samples.

In order to confirm this kind of vacancy ordering, kinematical diffraction patterns (*cf.* Figure 6) were calculated based on the structure model for oxygen-deficient trigonal Ba(Ti^4+^_1/3_Ti^3+^_2/3_)O_2.67_ given by [27]. The data for BaTiO_3_ in the cubic setting were taken from [29]. Apart from a small deviation of the average lattice parameters of the nanocomposite in comparison to bulk BaTiO_3_ (splitting of the 222 reflection; *cf.* Figure 5), the simulated SAED patterns (Figure 6) agree well with the experimental ones. This may suggest a close analogy between oxygen vacancy ordering in reduced BaTiO_3_ and that in our BaTiO_3_-BiFeO_3_ composite thin films. Figure S11 compares the calculated oxygen vacancy ordered “defective” BaTiO_3_ electron diffraction pattern with that of pseudocubic “stoichiometric” BaTiO_3_. The SAED image taken from the 0.25-mbar composite film depicted in Figure S10 confirms again the BaTiO_3_-type structure of the entire BaTiO_3_-BiFeO_3_ composite film.

### 2.3. Magnetoelectric Voltage Coefficients

**Figure 7:**
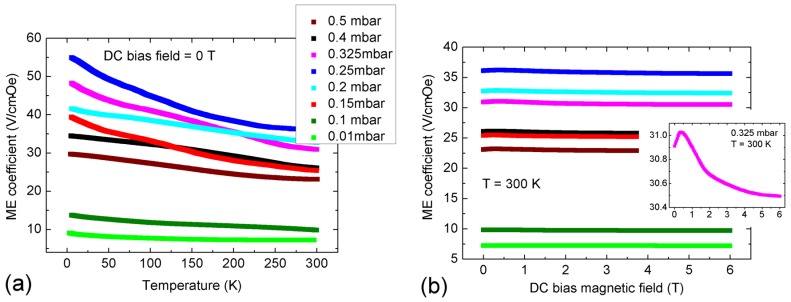
Magnetoelectric voltage coefficient α_ME_ of BaTiO_3_-BiFeO_3_ composite thin films in dependence of (**a**) temperature and (**b**) the DC bias magnetic field, for the indicated oxygen partial pressures during the growth of the composites. The legend of (a) is valid for both (a) and (b). The DC bias dependence of α_ME_ in (b) is generally weak with a local maximum around 0.5 T. The inset is an expanded view of α_ME_(H_DC_) of the 0.325 mbar sample G5558c, compare Table 1. For additional field-dependent α_ME_ graphs, see the Supplementary Materials, Figure S12.

Figure 7a,b shows the magnetoelectric voltage coefficient α_ME_ of the BaTO_3_-BiFeO_3_ composite thin films as a function of temperature and direct current (DC) bias magnetic field (H), respectively, for the indicated oxygen partial pressures during PLD growth. In these experiments, the measured electric field (E) is oriented parallel to the applied alternating current (AC) and DC H-fields. During the measurements, the AC H-field was kept constant, and we did not apply any stress nor an additional E-field to the samples. In the temperature dependencies, the DC H-field was also constant. For more details, see the Experimental Section.

The magnetoelectric coupling of the composite films increases with decreasing temperature, in contrast to that of our recently-reported BaTO_3_-BiFeO_3_ superlattices consisting of 15 pairs of stacked thin BaTiO_3_ and BiFeO_3_ layers; see [22]. We assume that in superlattice heterostructures with clearly separated phases, as reported in [11,22,23], the magnetoelectric coupling is dominated by magnetostrictive-piezoelectric interaction coupled via strain at the interfaces of both phases. In purely strain-mediated coupling of the piezoelectric (electrostrictive, ferroelectric) phase to the magnetostrictive (magnetoelastic, ferromagnetic) phase, the coupling should decrease with decreasing temperature due to the temperature dependence of magnetostrictive and piezoelectric coefficients. Because the temperature dependence of α_ME_ of the BaTO_3_-BiFeO_3_ composite thin films is opposite in tendency (Figure 7a), we expect the participation of an additional coupling mechanism, for example via charge; see the further discussion below. However, the temperature dependence of magnetoelectric coupling is not yet fully understood. The ferroelectric and magnetic phase transitions of undoped and Ba-doped BiFeO_3_ at high temperatures are reported in [30].

**Figure 8:**
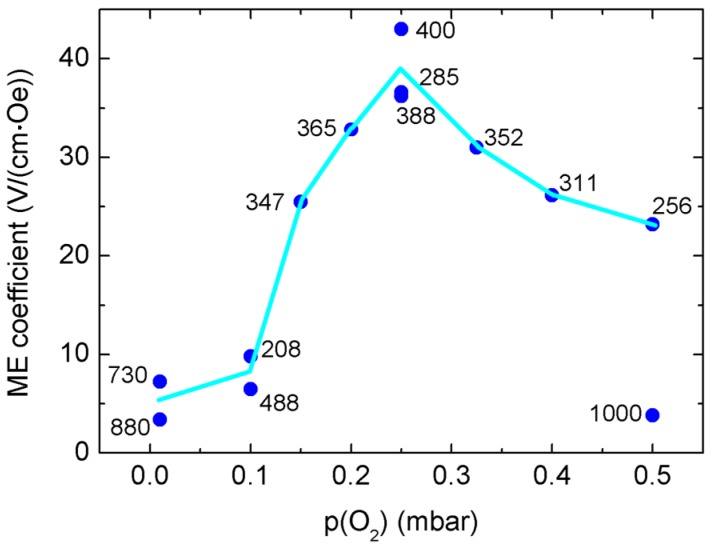
Magnetoelectric voltage coefficients α_ME_ of BaTO_3_-BiFeO_3_ composite thin films on SrTiO_3_:Nb(001) measured at 300 K and at the DC bias magnetic field with the maximum α_ME_. The number at each data point is the composite film thickness as determined from STEM images of cross-sections prepared by focused ion beam (see the Supplementary Materials, Figures S14 and S15). The line is drawn to guide the eye only. Using the film thicknesses, structural details of the samples (Table 1) can be assigned to the α_ME_ values.

In Figure S13, we show additionally typical ferroelectric hysteresis loops of two series of composite films grown simultaneously to that used for the magnetoelectric measurements in Figure 7 and Figure 8. Almost all samples show ferroelectric switching peaks in the current-voltage (I-V) characteristics used for calculation of the polarization-electrical field (P(E)) hysteresis loops. However, some samples show distorted I–V characteristics and P(E) loops, as SrTiO_3_:Nb substrates with different Nb contents of 0.1% and 0.5% were partly used for the samples called “b” and “d”, respectively; see Figure S13. Too high Nb content might introduce a barrier layer at the interface to the substrate, which results in rectifying, *i.e.*, Schottky behavior concerning the unavoidable leakage current through the films. However, high magnetoelectric coupling seems to correlate clearly with high saturation polarization, as the 0.25 mbar sample shows both the highest α_ME_ and the highest saturation polarization.

In our recent investigation of BaTiO_3_-BiFeO_3_ superlattices, we found anomalies in the temperature dependence of α_ME_ around the phase transition from tetragonal to orthorhombic BaTiO_3_ [22]. Furthermore, it is well known that strain in thin films may shift and remarkably broaden magnetic and ferroelectric transitions. For example, strain-induced shifts of the ferromagnetic Curie temperature of up to 19 K were found in La_0.7_Sr_0.3_MnO_3_ films [31]. The intrinsic piezoelectric coefficient *d*_31_, as well as the relative permittivity of Pb(ZrTi)O_3_ clearly decrease with decreasing temperature, while remanent polarization and the coercive field increase [32]. Because also magnetostriction decreases with decreasing temperature, the generally increasing α_ME_ with decreasing temperature cannot be explained by strain coupling alone. Rather, charge-related coupling mechanisms may play also a role here for our composites. Spurgeon *et al.* report direct local measurements of strain- and charge-mediated magnetization changes in the La_0.7_Sr_0.3_MnO_3_/PbZr_0.2_Ti_0.8_O_3_ system, which can be tuned by the manganite and ferroelectric layer thicknesses [33]. In contradiction to the chemically almost homogeneous composite films reported here, our superlattices consisting of clearly separated BaTiO_3_ and BiFeO_3_ films with a thickness of a few nm show the opposite temperature dependence of α_ME_ down to about 100 K and only below an increasing α_ME_; compare [22].

Figure 8 shows directly the dependence of α_ME_ on oxygen partial pressure during growth, for 300 K and at the DC-field of maximum α_ME_, *i.e.*, between 0 and 1 T. We found a reproducible maximum of α_ME_ around a 0.25-mbar growth pressure, and for composite thicknesses of 200–400 nm. Thicker composite films seem to show lower α_ME_ values; see Figure 8. The composite film thicknesses were determined directly and precisely from focused ion beam (FIB)-prepared cross-sections: see Figures S14 and S15. In [25], we have measured the trigonal distortion of oxygen tetrahedra of oxygen-deficient, weakly-Mn-doped ZnO thin films. Lower oxygen partial pressure in PLD growth is clearly correlated to increasing density of oxygen vacancies, which is accompanied by higher structural distortions, expressed locally by increasing variation of bond distances and rocking curve widths, *i.e.*, film mosaicity [25].

However, in contrast to the BaTiO_3_-BiFeO_3_ superlattices [22], we do not find a clear correlation of α_ME_ and the ω-related widths of the $\bar{1}03$ RSM lattice points; see Table 1 and Figure 3. The lowest growth pressures result in the narrowest XRD peak widths, *i.e.*, the smallest tilt mosaicity of the composite films. This behavior is again contradictory to the (BaTO_3_-BiFeO_3_) × 15 superlattices, where we found strongly increasing (001) ω-broadening with both decreasing growth pressure and α_ME_ [22]. The contradictory temperature dependencies of the α_ME_ of composites and superlattices and their sensitivity to external growth parameters, such as oxygen supply and layer geometry, indicate a complex interplay of strain- and charge-mediated magnetoelectric coupling, which requires further extensive efforts to become clearer in the future.

## 3. Experimental Section

The 67 wt % BaTiO_3_-33 wt % BiFeO_3_ composite films were grown by PLD from mixed phase targets with the above-mentioned composition, using a KrF excimer laser and a 10-cm target-to-substrate distance. The optimum growth temperature was 680 °C; there, the films are highly crystalline. The PLD oxygen partial pressure was controlled in between 0.01 and 0.5 mbar. In our PLD approach, four very similar films were grown simultaneously using a multi-substrate holder for epi-polished SrTiO_3_(001) and SrTiO_3_:Nb(001) with 0.1% and 0.5% Nb content and MgO(001). The SrTiO_3_ and SrTiO_3_:Nb substrates were HF etched and annealed prior to deposition to achieve a monolayer-terraced Ti-terminated surface. By XRD 2θ-ω scans, minor changes of the out-of-plane lattice parameter in dependence of the used substrate were found; see Ref. [11]. The conducting SrTiO_3_:Nb substrates are required for the determination of ME voltage coefficients together with Pt top contacts of various diameters to get a thin film capacitor structure. The film thickness was determined directly by STEM on embedded cross-sections exposed by focused ion beam (FIB); see the Supplementary Materials, Figures S14 and S15. For these STEM images with lower resolution, a field emission scanning electron microscope (FEM) FEI NOVA Nanolab 200 (FEI Europe Nano Port, Eindhoven, The Netherlands) was used. We adjusted the number of laser pulses in PLD to compensate partially the effect of background pressure on growth rate and to achieve a thickness of 250–400 nm for all applied oxygen partial pressures; see Figure 8. Thicker films stem from earlier growth runs. With that, we monitor film thickness effects on magnetoelectric coupling. More details of the growth process, as well as the structural, ferroelectric and magnetic response of the samples can be found in [11]. For a recent description of the state of the art of PLD growth, see also the Special Issue “25 years of pulsed laser deposition” [34].

XRD wide-angle 2θ-ω scans, reciprocal space maps around the symmetric 001 and 002 and the asymmetric $\bar{1}03$ lattice points of the SrTiO_3_:Nb(001) substrates were measured using a PANalytical X'pert PRO MRD (PANalytical B.V., Almelo, The Netherlands) with Cu Kα_1/2_ radiation from a parabolic mirror and a PIXcel^3D^ array detector (PANalytical B.V.) with an electronically controlled receiving slit width.

Free-standing cross-sections for STEM were prepared from two samples, *i.e.*, the 67 wt % BaTiO_3_-33 wt % BiFeO_3_ composite films grown at either 0.25 mbar or 0.01 mbar oxygen partial pressure on MgO(001). Cross-sections about 100 nm thick were extracted using the FIB of the above-mentioned FEM Nanolab 200. Further thinning up to 200 kV electron transparency was done by Ar^+^ ion milling in a Gatan PIPS instrument (Gatan, Inc., Pleasanton, CA, USA). STEM was carried out in a Philips CM-200 STEM (FEI Europe Nano Port) with a super-twin objective lens (point resolution of 0.23 nm). SAED patterns were taken from selected regions of the cross-sections. The distribution of elements was determined by EDX mapping (EDAX detector system). A weak Bi-deficit (more pronounced for the 0.01 mbar sample) was found in the composite films due to the use of a stoichiometric PLD source target and the lower sticking of Bi-species at the heated substrate surface; see Figures S6 and S9 for quantitative EDX analyses of the two investigated samples in the Supplementary Materials. Kinematical electron diffraction patterns were simulated using the JEMS software package (École Polytechnique Fédérale de Lausanne, Villigen, Switzerland) [35]. The structure data (not refined) for “reduced” BaTiO_3_ required for simulation were taken from [27] with data code ICSD 54785.

The magnetoelectric voltage coefficients α_ME_ were measured in a Quantum Design physical property measurement system (PPMS). The AC voltage induced inside the capacitor structure with the composite film as the medium in between the two electrodes is measured with a lock-in amplifier SR 830 (Stanford Research Systems, Inc., Sunnyvale, CA, USA) in response to a small AC magnetic field with a fixed frequency of 1 kHz and a field strength H_AC_ of 10 Oe (1 Oe = 10^3^/4π·A·m^−1^). Both AC and possible additional DC magnetic fields are applied in the out-of-plane direction of the capacitor, *i.e.*, along the ME voltage in longitudinal geometry. For more details and corresponding ME measurements on BiFeO_3_-based bulk samples and BiFeO_3_ films, see [36] and [23], respectively.

Ferroelectric hysteresis loops were measured using a thin film analyzer TF 2000 HS (aixACCT Systems GmbH, Aachen, Germany) in dynamic hysteresis mode with frequencies of 100 Hz up to 1 kHz, without or with leakage current compensation. Triangular excitation pulses were applied in the dynamic sequences. The diameter of Pt top contacts was usually 225 μm.

## 4. Conclusions

BaTiO_3_-BiFeO_3_ composite thin films with a mixing ratio of the PLD source target of 67 wt % BaTiO_3_ and 33 wt % BiFeO_3_ and a total film thickness of typically 350 nm show a clear dependence of magnetoelectric voltage coefficient α_ME_ on oxygen partial pressure during growth. Three composite films grown at 0.25 mbar show the highest α_ME_ values of 36–43 V/(cm·Oe) at 300 K. α_ME_ decreases for both lower and higher growth pressure, due to increasing oxygen deficiency and increasing crystallite size, respectively. The composite films are grown epitaxially on SrTiO_3_:Nb(001) substrates and exhibit increasing out-of-plane strain with decreasing PLD growth pressure in the range from 0.5 mbar down to 0.01 mbar. Because the out-of-plane lattice parameters of BaTiO_3_ and BiFeO_3_ are very close together, the corresponding XRD peaks of the two phases could not be resolved. Therefore, the detailed microstructure of the composite films on the atomic length scale has been unknown up to now.

However, STEM micrographs and SAED patterns taken from different regions of cross-sections of the composite films indicate an oxygen vacancy superstructure, which arises from vacancy ordering on the {111} planes of the pseudocubic BaTiO_3_-type structure of the composite films. The intensity of the additional superstructure reflections seems to correlate to the oxygen growth pressure of the two investigated samples. This means that the sample grown at lower (0.01 mbar) pressure seems to show a more pronounced oxygen vacancy superstructure in comparison to the sample grown at higher (0.25 mbar) p(O_2_). In our previous work on oxygen-deficient ZnO thin films [25], we found a clear correlation of the PLD growth pressure and oxygen deficiency of films, which goes along with the structural distortions of the cation-anion octahedra.

Contrary to our recently investigated (BaTiO_3_-BiFeO_3_) × 15 superlattices [11,22,23], α_ME_ shows an increasing behavior with decreasing temperature, which cannot be explained by strain-mediated ME coupling of piezoelectric and magnetostrictive phases in the composite films alone. Rather, charge-mediated ME coupling may play a role here. Further research is planned to understand the observed temperature and DC magnetic field dependencies of α_ME_ more clearly to be able to design magnetoelectric composites with clear application perspectives.

## Supplementary Materials

The following are available online at www.mdpi.com/1996-1944/9/1/44/s1, Figures S1 to S15. We show additional RSMs and XRD scans (Figures S1–S3), and additional transmission electron microscopy (TEM) images and energy dispersive X-ray spectroscopy (EDX) analyses of two selected BaTiO_3_ (67%)-BiFeO_3_ (33%) composite samples grown at 0.01 mbar (Figures S4–S7), and at 0.25 mbar (Figures S7–S11). Furthermore, we show further details of magnetoelectric voltage coefficients (Figure S12), corresponding ferroelectric hysteresis loops (Figure S13), as well as FIB extractions and low-resolution scanning transmission electron microscopy (STEM) images from the field emission scanning electron microscope (FEM) (Figures S14 and S15).
