# Supplementary material for: Correlation of High Magnetoelectric Coupling with Oxygen Vacancy Superstructure in Epitaxial Multiferroic BaTiO3-BiFeO3 Composite Thin Films
Source: Materials (Basel). 2016 Jan 13;9(1):44. doi: 10.3390/ma9010044 (PMC5456545; doi:10.3390/ma9010044)
Supplement: Supplementary file 1 [file materials-09-00044-s001.pdf]

Supplementary Materials: Correlation of High Magnetoelectric Coupling with Oxygen Vacancy Superstructure in Epitaxial Multiferroic $BaTiO_3$-$BiFeO_3$ Composite Thin Films

Michael Lorenz, Gerald Wagner, Vera Lazenka, Peter Schwinkendorf, Michael Bonholzer, Margriet J. Van Bael, André Vantomme, Kristiaan Temst, Oliver Oeckler and Marius Grundmann

Here we show additional RSMs and XRD scans (Figures S1–S3), and additional transmission electron microscopy (TEM) images and energy dispersive X-ray spectroscopy (EDX) analyses of two selected $BaTiO_3$ (67%)-$BiFeO_3$ (33%) composite samples grown at 0.01 mbar (Figures S4–S7), and at 0.25 mbar (Figures S7–S11). Furthermore, we show further details of magnetoelectric voltage coefficients (Figure S12), corresponding ferroelectric hysteresis loops (Figure S13), as well as FIB extractions and low-resolution scanning transmission electron microscopy (STEM) images from the field emission scanning electron microscope (FEM) (Figures S14 and S15).

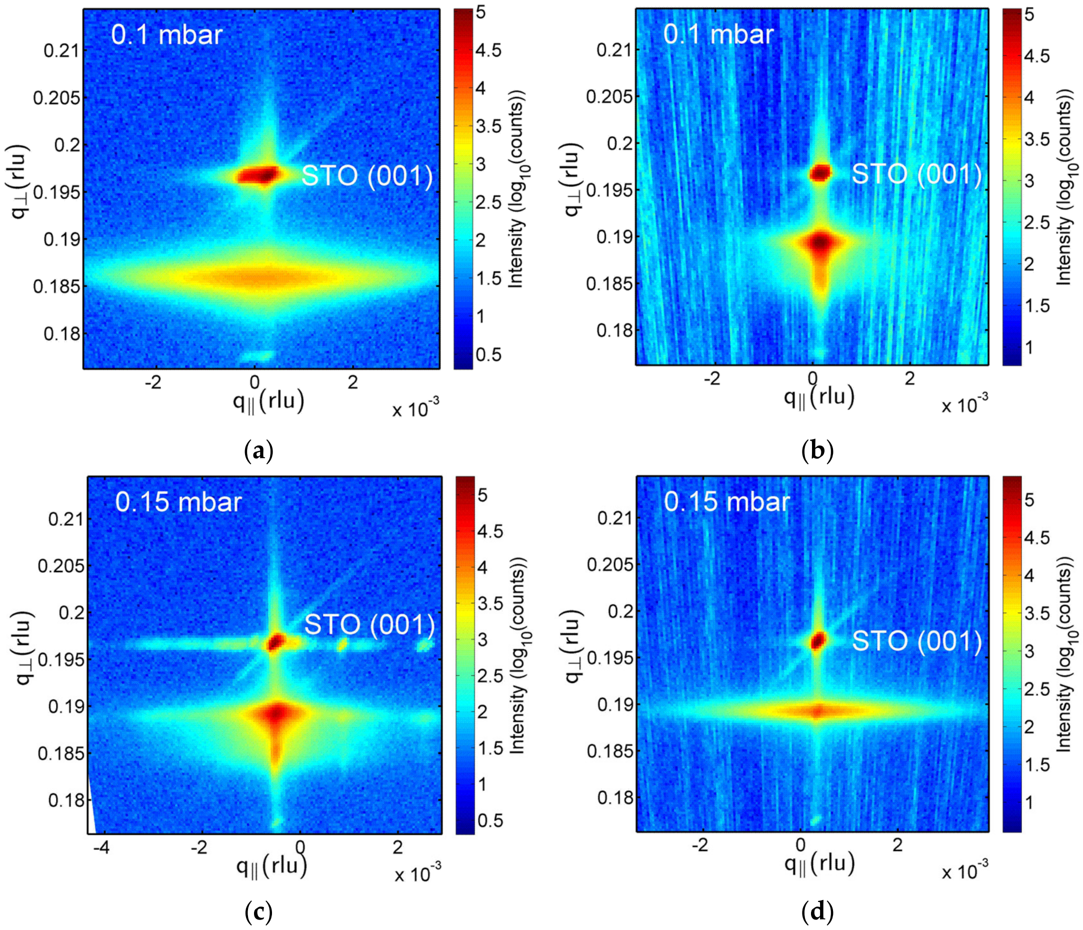

Figure S1. Effect of substrate mosaizity of samples (**a**) G5562b grown at 0.1 mbar, and (**c**) G5561b grown at 0.15 mbar on composite film mosaizity, as shown in the left RSMs. The samples (**b**) G5562d and (**d**) G5561d shown right are grown simultaneously with the left samples (**a**) and (**c**), respectively. Therefore, the differences of film reflections are attributed to the mosaizity and multi-domain structure of STO substrates, *i.e.*, left side substrate peaks in (**a**) and (**c**) are broadened or split horizontally, respectively. More structural data of these four samples are listed in Table 1. STO stands for $SrTiO_3$.

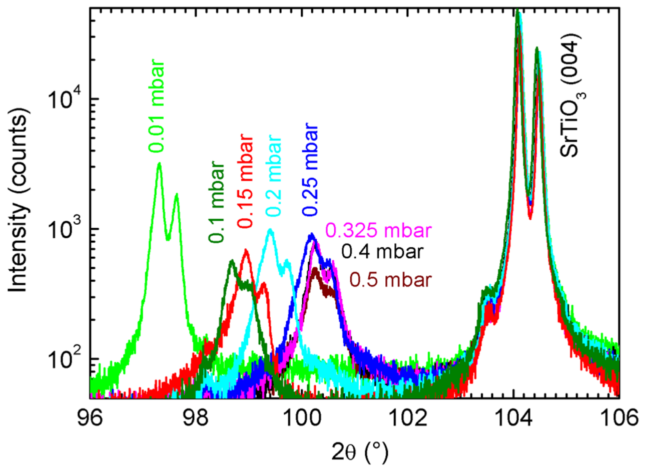

Figure S2. XRD 2θ-ω scans of $BaTiO_3$-$BiFeO_3$ composite thin films grown on $SrTiO_3$:Nb (001) at the indicated oxygen partial pressures. Note the $K\alpha_{1/2}$ splitting with intensity ratio 2:1 of each peak. These 004 composite peak positions are used for calculation of the out-of-plane lattice constants in Figure 1b and Table 1.

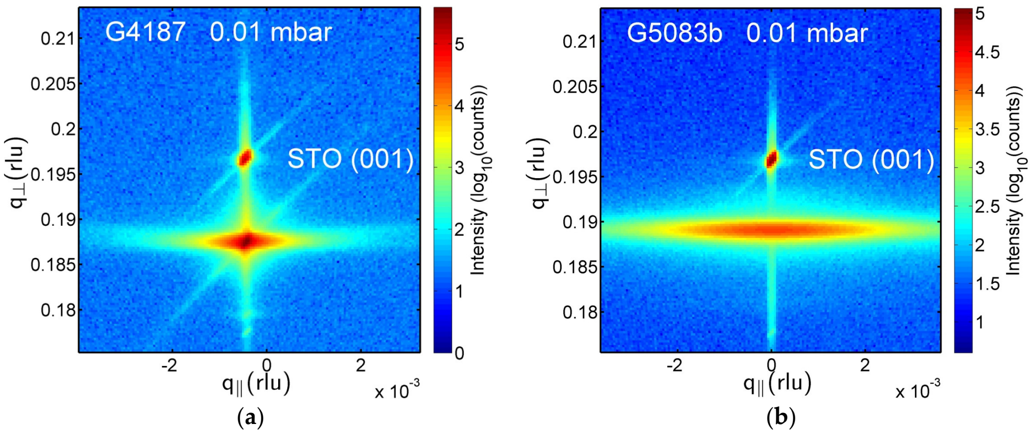

Figure S3. Effect of $BaTiO_3$-$BiFeO_3$ mixing ratio as visible in RSMs around $SrTiO_3$(001). (**a**) is a 67 wt %-33 wt % composite, and (**b**) a 33 wt %-67 wt % composite film, respectively, both grown at 0.01 mbar. Horizontal film peak width (FWHM) as a measure of tilt mosaizity is (**a**) 0.069°, and (**b**) 0.600°. See [11] for more details.

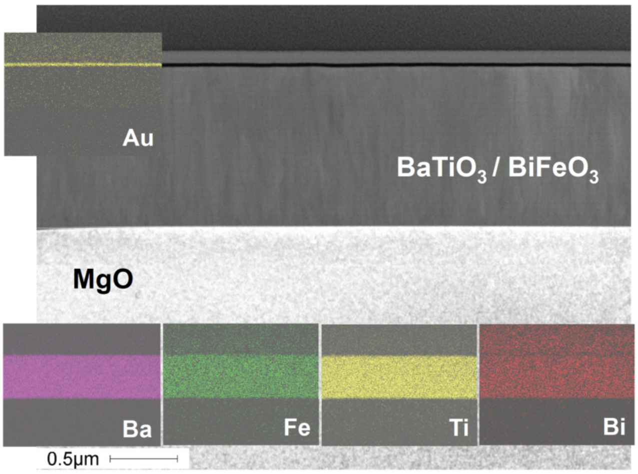

Figure S4. STEM dark-field image of sample G4188 grown at 0.01 mbar; (110) cross section. The EDX maps show the homogeneous distribution of indicated elements in the cross section.

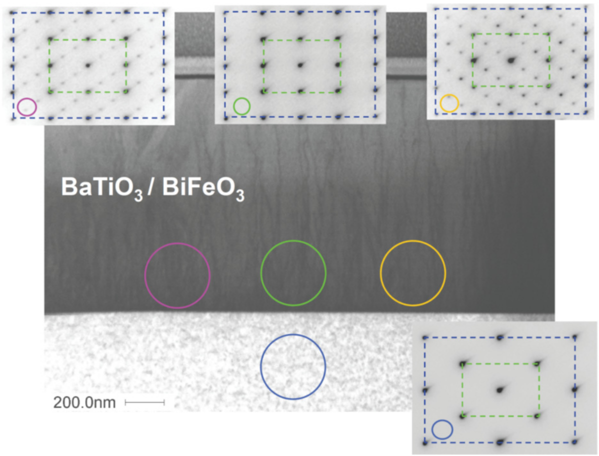

Figure S5. STEM bright-field of sample G4188 grown at 0.01 mbar; (110) cross section. The SAED images stem from the encircled regions in the cross section.

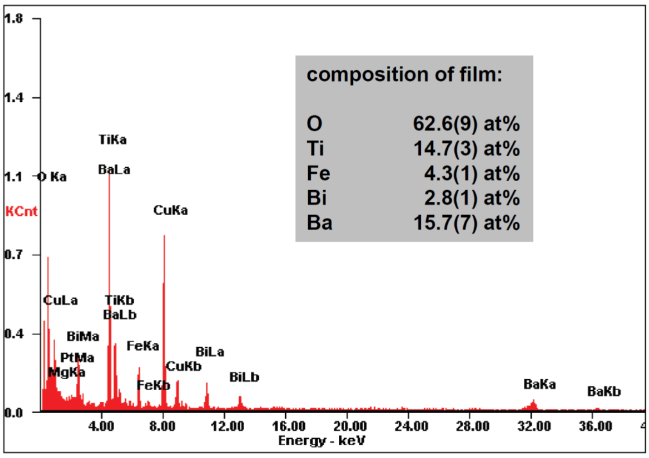

Figure S6. EDX measurements of the composite film G4188 grown at 0.01 mbar. Note, the Cu-peak stems from the copper sample holder. There is no significant change of composition inside the layer, *i.e.*, the layer is homogeneous, see EDX maps. The inset shows the element concentrations as average of 5 measurements together with standard deviations. Note the increased Bi deficit with the lower growth pressure in comparison to Figure S9.

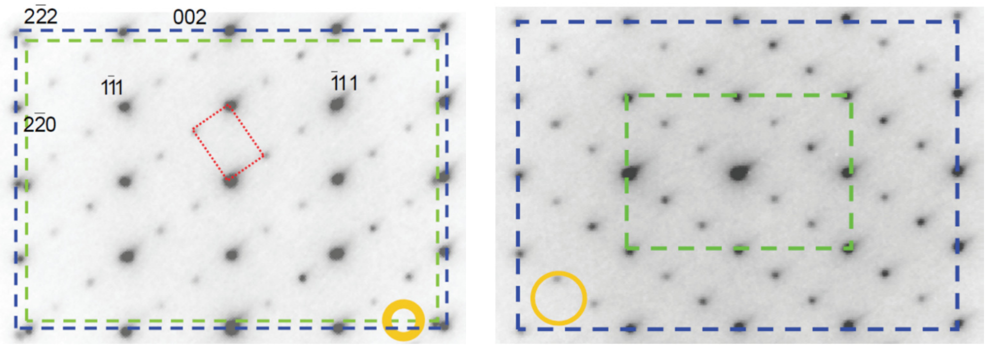

(**a**) (**b**)

Figure S7. Comparison of SAED images of composite films taken from (**a**) G4184 grown at 0.25 mbar, and (**b**) G4188 at 0.01 mbar, see Figures 5 and S5, respectively. The additional reflections due to the oxygen vacancy superstructure seem to be visually more intense in (**b**), *i.e.*, for the sample grown with higher oxygen deficiency at 0.01 mbar. However, a detailed quantitative comparison requires much more effort in careful calibration of electron diffraction intensities in the SAED images.

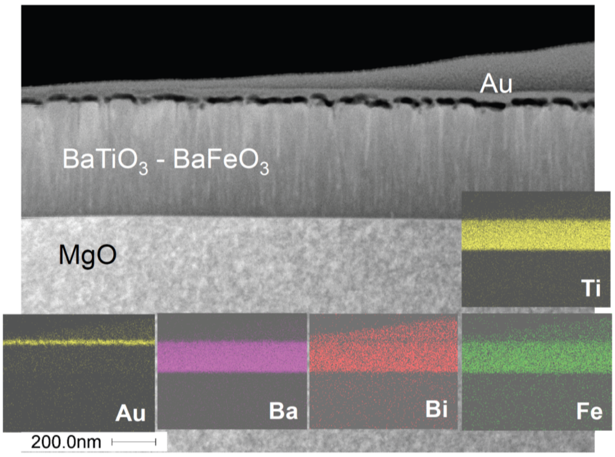

Figure S8. STEM dark field image of sample G4184 grown at 0.25 mbar; (110) cross section. The EDX maps of selected elements show homogeneous distributions within the composite film cross sections.

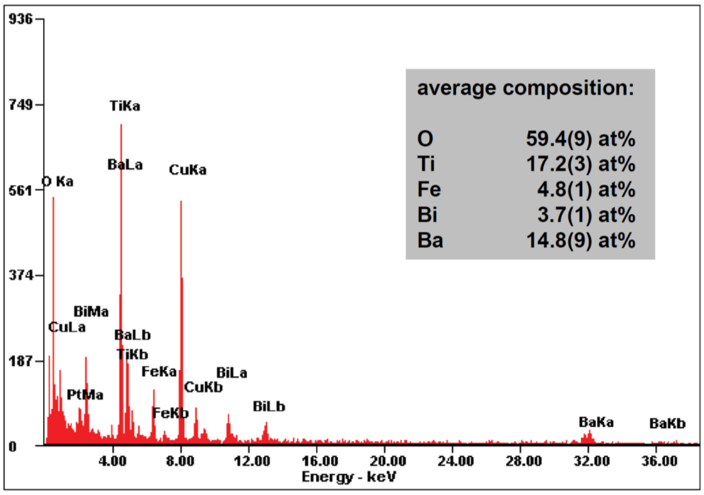

Figure S9. EDX spectrum of the composite film G4184 grown at 0.25 mbar. Note, the Cu-peak stems from the copper sample holder. There is no significant change of composition inside the layer. The inset gives the average composite composition of 5 measurements together with standard deviations.

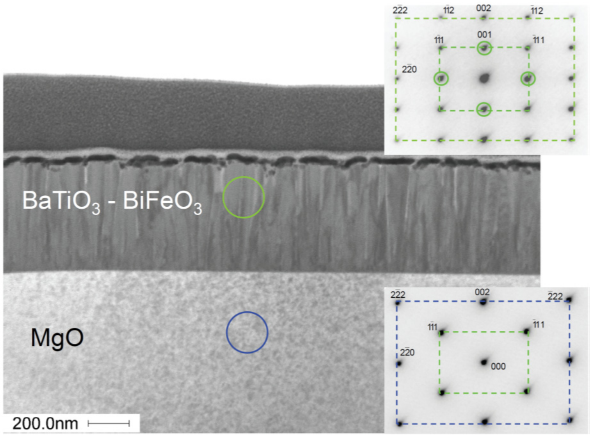

Figure S10. STEM dark field image of sample G4184 grown at 0.25 mbar; (110) cross section. The SAED images are taken from the encircled regions in the cross section (green circle: middle of composite film, blue circle: MgO substrate only). In the top right SAED image, the indicated green spots confirm the $BaTiO_3$-type structure of the entire film. These are forbidden for MgO as seen bottom right.

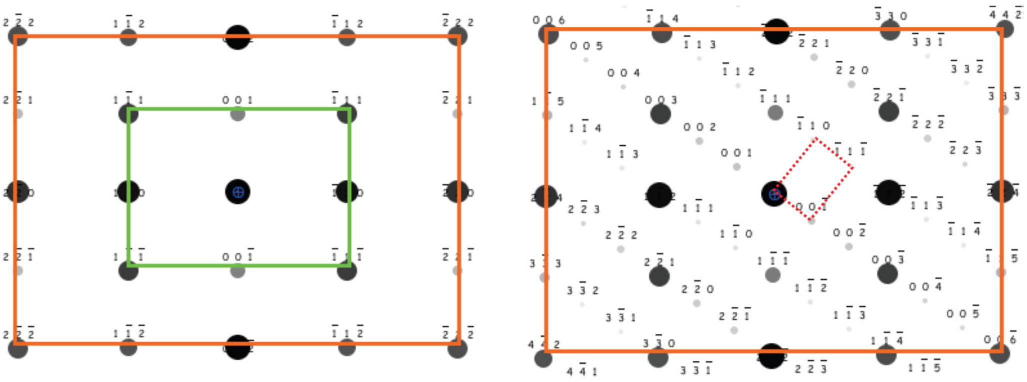

(**a**) (**b**)

Figure S11. Calculated electron diffraction pattern of (**a**) pseudocubic "stoichiometric" and (**b**) oxygen vacancy ordered "defect" $BaTiO_3$. The vacancy ordering appears on $(1\bar{1}1)_c$ or $(0001)_h$, *i.e.*, the pseudocubic unit cell is tripled, see tilted dotted red rectangle.

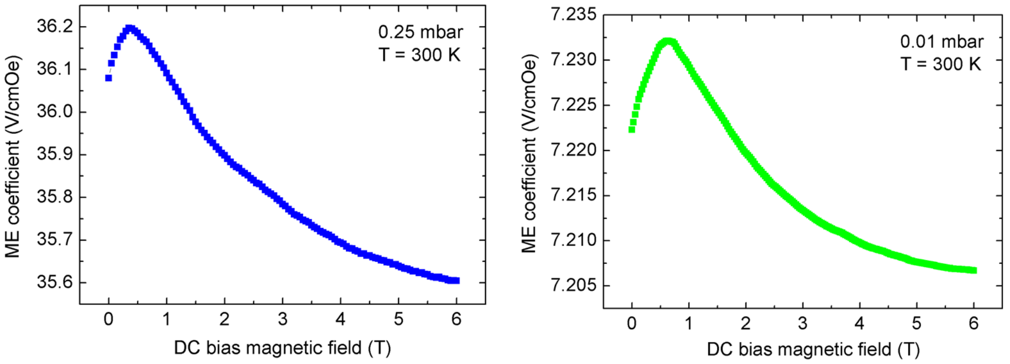

(**a**) (**b**)

Figure S12. Magnetoelectric voltage coefficients α_{ME} in dependence on DC bias magnetic field of two selected composite film samples grown at (**a**) 0.25 mbar, and (**b**) 0.01 mbar oxygen partial pressure during PLD. Note the expanded α_{ME} scale in comparison to Figure 7b.

	**b**	**d**
G5556 (0.5 mbar O_2)	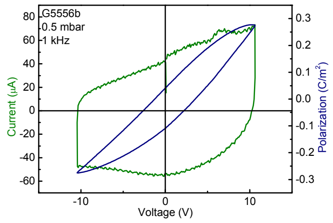	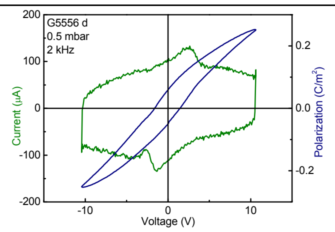
G5557 (0.4 mbar O_2)	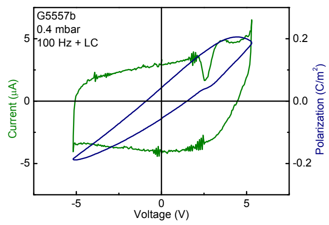	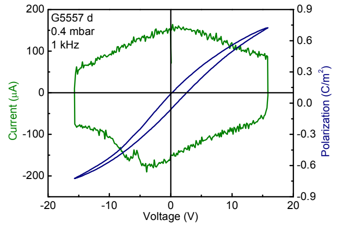
G5558 (0.325 mbar O_2)	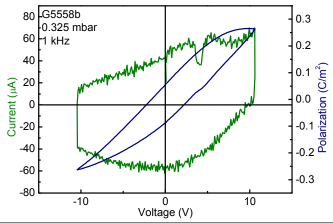	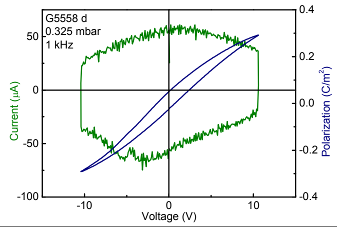

Figure S13. *Cont.*

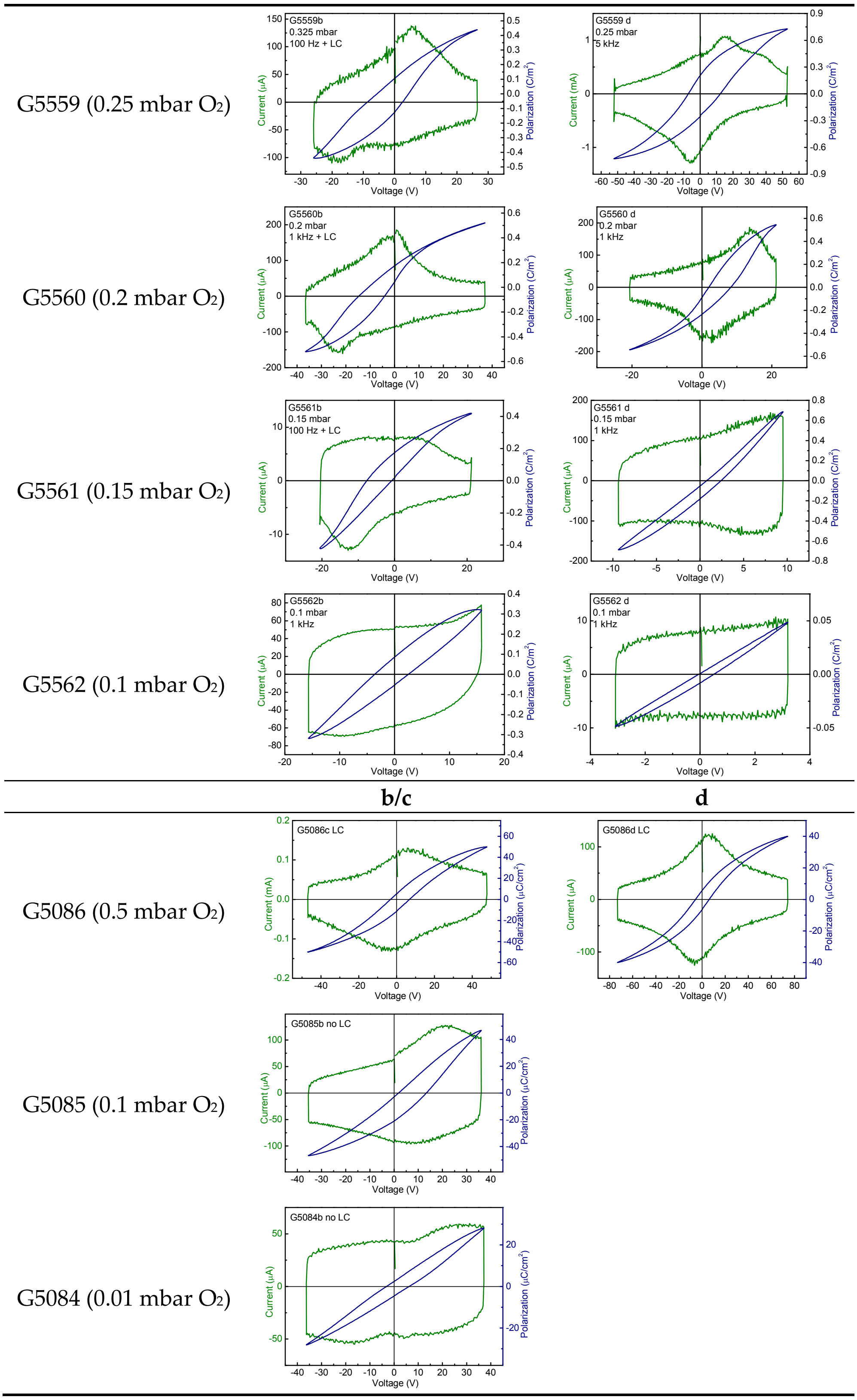

Figure S13. Ferroelectric hysteresis loops (blue curves with right scales) and I-V curves (green curves with left scales) of two series G5556-62 (previous side), and G5084-86 (above) of BaTiO3-BiFeO3 (67%-33%) composite films grown from 0.5 mbar (top) to 0.01 mbar (bottom). Measurement frequency was in between 100 Hz and 5 kHz. The columns **b**, **b/c**, and **d** refer to different samples (note the letters at the end of sample Nos.) grown simultaneously on different SrTiO3:Nb (001) substrates, respectively. The composite film G5559d grown at 0.25 mbar shows FE hysteresis loop with highest saturation polarization and coercive field. Note, for the unit of polarization 1 C/m^2 = 100 μC/cm^2.

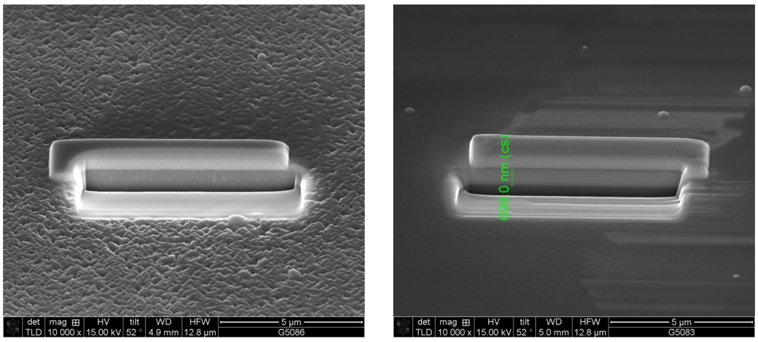

(**a**) (**b**)

Figure S14. FEM image of finished FIB extraction of film cross sections of samples grown at (**a**) 0.5 mbar, and (**b**) 0.01 mbar, for precise determination of geometrical film thickness, see Figure S15.

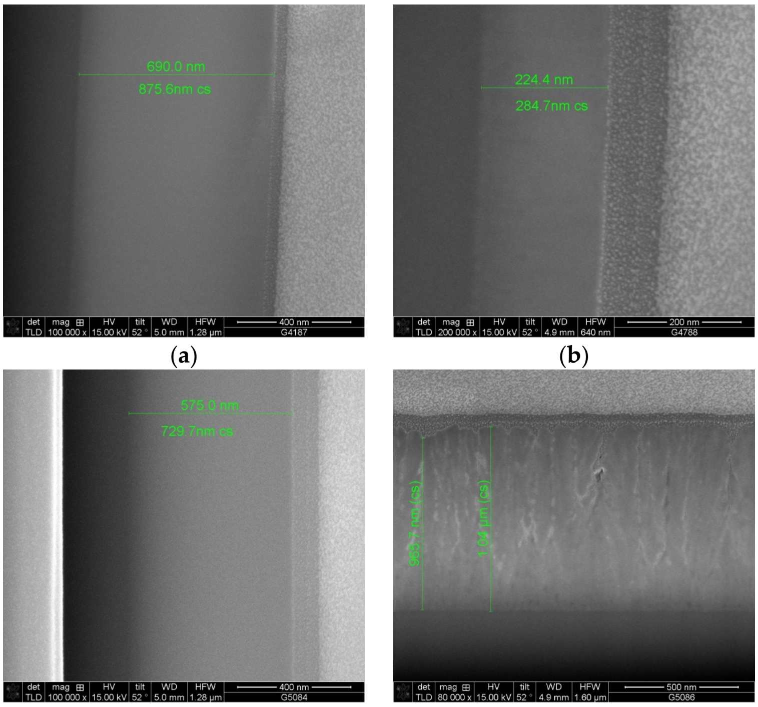

(**c**) (**d**)

Figure S15. FEM images of $BaTiO_3$-$BiFeO_3$ composite films on $SrTiO_3$:Nb 0.5%, with granular Pt layer of about 100 nm on top, for samples (**a**) G4187, (**b**) G4788, (**c**) G5084, and (**d**) G5086. Only the scan-rotated images give the correct film thickness marked by "cs".
